# Photothermal drivers and climate-sensitivity windows of Lanzhou lily (*Lilium davidii* var. *unicolor*) phenology across three decades of warming in Northwest China

**DOI:** 10.3389/fpls.2026.1792696

**Published:** 2026-04-17

**Authors:** Xiaohui Ma, Ronghua Yue, Hua Li, Xinyi Liu, Yingtai Pei, Ruitao Zhao, Yamin Wu, Ling Jin

**Affiliations:** 1 Gansu Pharmaceutical Industry Innovation Research Institute, Gansu University of Chinese Medicine, Lanzhou, China; 2 State Key Laboratory of Cryospheric Science and Frozen Soil Engineering, Northwest Institute of Eco-Environment and Resources, Chinese Academy of Sciences, Lanzhou, China; 3 Gansu Xinglongshan National Nature Reserve Management Centre , Yuzhong, China

**Keywords:** biometeorology, *Lilium davidii* var. unicolor, photothermal control, plant phenology, semi-arid agriculture, sliding-window analysis

## Abstract

Plant phenology provides an integrative biometeorological indicator of how agroecosystems respond to climate variability and long-term change. The Lanzhou lily (*Lilium davidii* var. *unicolor*), a key edible–medicinal bulb crop in Northwest China, is cultivated in a temperate semi-arid region that has experienced pronounced warming and a concurrent decline in sunshine duration over recent decades. Using continuous field phenological observations (1995–2024) and daily meteorological records, we quantified long-term trends in four phenological metrics (emergence, full bloom, shoot senescence, and growing season length) and identified phase-specific climatic sensitivity windows via a sliding-window correlation approach. We then parameterized multiple regression models using the optimal windows to quantify the relative roles of temperature, sunshine duration, and precipitation. Over the 30-year period, emergence, full bloom, and shoot senescence advanced significantly (1.06, 1.23, and 1.69 days per decade, respectively), while growing season length shortened (0.67 days per decade). Temperature exerted a consistent advancing effect across all phases, whereas sunshine duration acted in the opposite direction by delaying full bloom and senescence. Precipitation contributed a minor, late-season influence on growing season length. The identified response windows (from early spring to late summer) provide a practical basis for biometeorological monitoring and phenology forecasting of this perennial bulb crop under ongoing climate change.

## 1 Introduction

The Lanzhou lily (*Lilium davidii* var. *unicolor*) is a perennial herb of the genus Lilium, and an endemic variety native to China. It is primarily cultivated in Lanzhou and surrounding areas of Gansu Province (Li et al., 2020a), where its unique biological traits, high edible and medicinal value, and strong adaptability to arid and semi-arid environments make it a representative economic and ecological crop in this region. The species is characterized by large, fleshy, pure-white bulbs with a naturally sweet taste, earning it the title of China’s only widely recognized “sweet lily” (Liao et al, 2025; Zhang et al., 2015). Its bulbs are rich in proteins, polysaccharides, amino acids, saponins, and essential minerals, which underpin traditional medicinal uses such as moistening the lungs, relieving coughs, and calming the mind (Satou et al., 1996). Modern pharmacological studies have confirmed that Lanzhou lily extracts possess multiple bioactivities, including antioxidant, antitumor, and immunomodulatory effects (Kang et al., 2022; Li et al., 2013; Sun et al., 2014). Ecologically, the Lanzhou lily grows on mountain slopes (Cao et al., 2024) at altitudes of 1,800–2,600 m under a temperate semi-arid climate, with a mean annual temperature of about 9.1°C and annual precipitation of 300–450 mm. The Lanzhou lily exhibits rapid responses to phenological changes, particularly to temperature variations (Tang et al., 2021; Cao et al., 2023). Even slight fluctuations in temperature can lead to measurable shifts in its phenological timing, resulting in either an advancement or delay of key growth stages.

Phenological phases represent the critical temporal rhythm in the growth (Piao et al., 2019; Wang et al., 2010) of the Lanzhou lily, exerting a pronounced effect on its yield and quality. Among the key stages, the Emergence Stage (ES) marks the onset of vegetative growth, and its timing determines the effective duration of the growing period (Geng et al., 2022). Earlier emergence extends the photosynthetic period, promotes the accumulation of assimilates, and enhances bulb biomass and individual yield (Zheng, 2011). The interval between the ES and the Full Bloom Stage (FBS) regulates the allocation of photosynthates and energy resources (Gough et al., 2010; Gougherty and Gougherty, 2018). When this interval shortens, premature flowering may lead to excessive nutrient allocation toward reproductive organs, thereby reducing nutrient transport to the bulb and ultimately constraining bulb expansion and quality formation (Zhang et al., 2010). Additionally, the phenological transition from spring to autumn is further influenced by the plant’s nutritional status (Fu et al., 2019b). The overall Growing Season Length (GSL) defines the effective duration of photosynthetic activity and is closely linked to final bulb size and nutrient composition, serving as a fundamental determinant of yield and quality. Therefore, accurately characterizing the phenological variation of the Lanzhou lily is crucial for developing climate-adaptive cultivation strategies and achieving high yield and superior quality under changing climate conditions.

Temperature is widely recognized as the primary climatic factor influencing the phenology of the Lanzhou lily (Fu et al., 2019a; Zhang et al., 2022; Shen et al., 2022). Rising air temperature has been shown to significantly advance spring phenophases in temperate plant species (Xu et al., 2021), while other studies suggest that climate warming may delay spring phenology under increasing winter temperatures due to reduced chilling accumulation and weakened dormancy release (Piao et al., 2019). In addition to temperature, sunshine duration is another key factor shaping phenological timing, whereas precipitation can indirectly affect spring and autumn phenophases by modifying soil moisture and thermal conditions (Peñuelas et al., 2004; Fu et al., 2014). Observations from the Lanzhou region show a marked warming trend over the past three decades, coupled with more frequent and intense heatwaves, heavy rainfall, and drought events (An et al., 2020). Such rapid climatic shifts are expected to exert substantial impacts on crop phenology (Yanxi et al., 2019). Previous studies have demonstrated that potato growth and development in the Loess Plateau of central Gansu are particularly sensitive to thermal anomalies (Yang et al., 2023). Similarly, in Gansu, the number of days with temperatures ≥30 °C during the spring wheat growing season has decreased (Wang et al., 2015) and the regreening phase of winter wheat has advanced significantly under warming (Wang et al., 2011). To date, research on this species has been dominated by short-term physiological or controlled-environment studies (Li et al., 2020b, Li et al., 2011), leaving a paucity of long-term, field-based evidence quantifying its phenological responses to climate variability. Consequently, it remains unclear how, and to what extent, the phenology of the Lanzhou lily has responded to such rapid climate change.

To address this knowledge gap, this study integrates 30 years (1995-2024) of continuous field phenological observations of Lanzhou lily with regional meteorological records. Using a combination of sliding-window correlation analysis and multiple linear regression, we identified phase-specific climatic sensitivity windows and quantified the relative associations of temperature, sunshine duration, and precipitation with interannual variation in key phenophases. Rather than constructing a process-based dynamical model, this study aims to provide statistically supported evidence for climate–phenology linkages and an empirical basis for future mechanistic modeling.

## 2 Materials and methods

### 2.1 Study area and observation site

**Figure 1:**
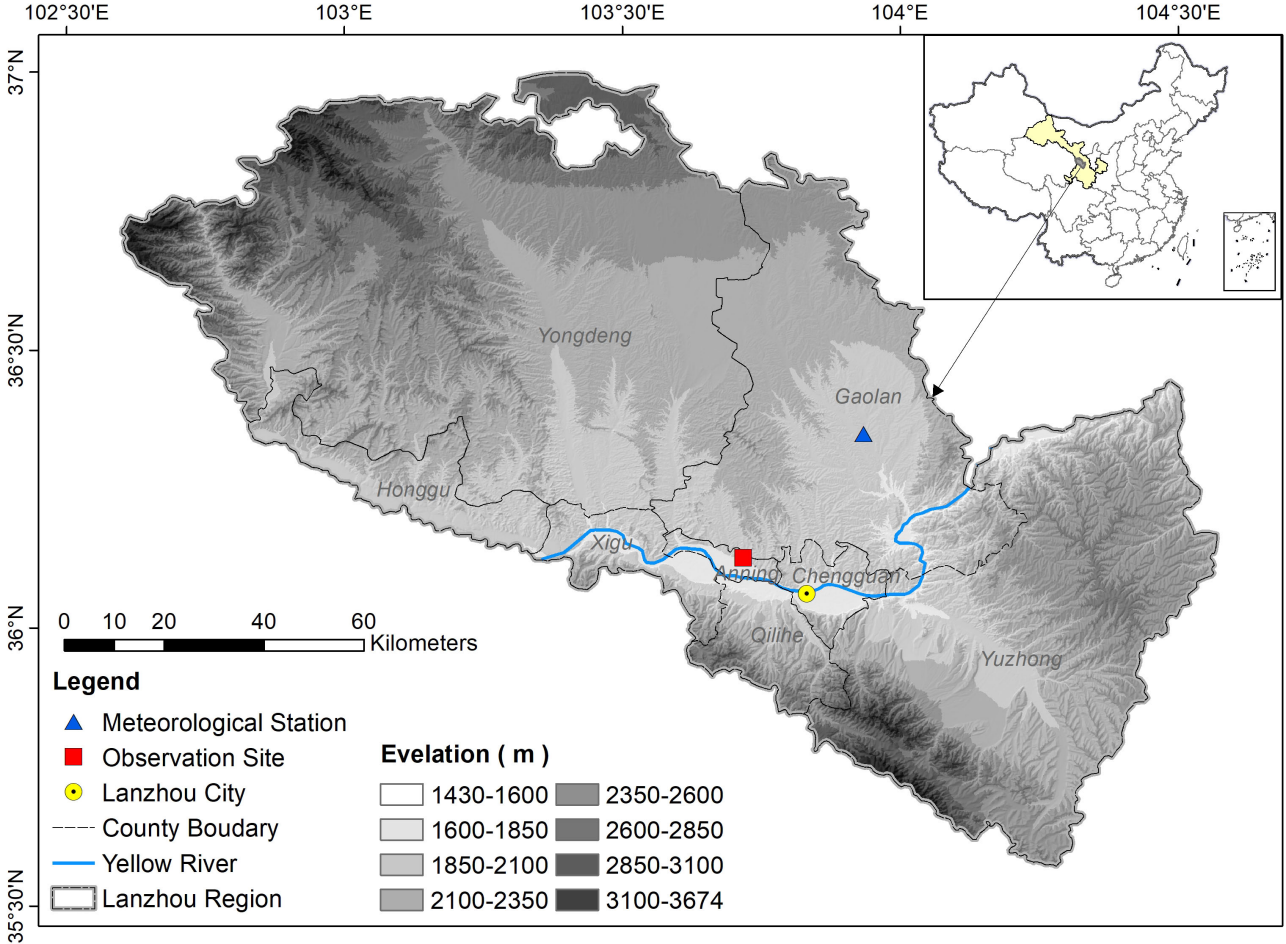
Location of the phenological observation site and meteorological station. The red rectangle indicates the field site where phenological observations of Lanzhou lily were recorded. The blue triangle shows the location of Gaolan meteorological station. The yellow dot represents the location of Lanzhou City, the blue curve shows the Yellow River, and the elevation increases from light to dark gray shading.

The phenological observation site of the Lanzhou lily was situated in Beishan Experimental Station in Anning District, Lanzhou, Gansu Province (103.72°E, 36.12°N, 1654.5 m a.s.l.) (**Figure 1**). The site lies within a typical temperate semi-arid continental climate zone, characterized by four distinct seasons, a mean annual temperature of 10.3 °C, annual precipitation of approximately 327 mm. The observation period spanned 1995-2024. The experimental site consists of seven lily plots, in which sprouting bulbs were transplanted on 2 April 1994 at an initial length of 7.5 ± 1 cm and a planting density of 38 plants m^-2^. The phenological observation plots were established in an open cultivation field, within an enclosed area of 53 m×67 m, located more than 100 m away from the adjacent woodland boundary, ensuring largely open-sky exposure for the plants. To ensure representativeness and exclude non-climatic noise, 20 healthy individuals were randomly selected and tagged in each of the seven plots annually (n = 140). Given the biological traits of the Lanzhou lily, bulbs were renewed and replanted every three years to maintain consistent physiological vigor throughout the 30-year period. Furthermore, management practices remained strictly consistent from 1995 to 2024: the site was entirely rain-fed (no irrigation), and a constant level of organic base fertilizer was applied annually. Weed control was performed manually to avoid competition without the use of chemical growth regulators. This standardized protocol ensures that the observed phenological shifts are primarily driven by climatic variability. The soil profile comprises an organic layer 13 ± 1.5 cm thick, underlain by a sandy-gravel substrate. This long-term, fixed observation site provides a robust foundation for investigating the adaptive growth patterns of the Lanzhou lily and its phenological responses to climate change.

Phenological observations of the Lanzhou lily were conducted in accordance with the Specifications for Agrometeorological Observation (China meteorological administration, 1993) issued by the China Meteorological Administration (CMA). Daily field observations were conducted at fixed monitoring plots by trained personnel at consistent times each day. The onset of each phenophase was defined as the date on which ≥50% of sampled plants met the corresponding phenological criterion. Four key phenological stages were monitored and recorded to the exact calendar day: (1) Emergence Stage (ES): defined as the date when the underground bulb has sprouted and the seedling has just emerged above the soil surface (≥50% of plants). (2) Full Bloom Stage (FBS): the date when ≥ 50% of plants reached full flowering. (3) Shoot Senescence Stage (SSS): the date when ≥50% of aboveground shoots showed visible yellowing or withering. (4) Growing Season Length (GSL): the number of days between ES and SSS. All phenological dates were converted into Day of Year (DOY) format, representing the number of days elapsed since 1 January of each year. Interannual variations in each phenophase and GSL were analyzed to characterize long-term phenological dynamics of ES, FBS, SSS, and GSL in the Lanzhou lily.

### 2.2 Meteorological data

Meteorological data were obtained from the Gaolan Meteorological Station (36.35° N, 103.93° E; 1667.2 m a.s.l.) (**Figure 1**), the nearest national basic meteorological station to the Lanzhou lily observation field, covering the period from 1995 to 2024. Its elevation, terrain, and soil conditions closely match those of the Lanzhou lily observation field. Given its proximity, the Gaolan National Meteorological Station provides a consistent regional-scale meteorological forcing for the study period. Because the phenology plots are located in open fields (>100 m from the woodland boundary), they are expected to capture broadly comparable weather variability, while we interpret station-based variables—particularly sunshine duration (Ssd)—as regional proxies of background sky conditions rather than exact plot-level irradiance. The dataset comprised daily records of seven key meteorological variables: mean air temperature (T_m_), maximum temperature (T_max_), minimum temperature (T_min_), relative humidity (RH), precipitation (Pre), sunshine duration (Ssd), and accumulated temperature above 0 °C (∑T_0_). Missing or abnormal meteorological values (<0.1% of the total observations) were corrected using adjacent-day mean interpolation to maintain the continuity of the daily climate time series. Because the proportion of corrected values is extremely small and the climatic variables used in the analysis are aggregated over multi-day windows (15–100 days), the influence of individual interpolated values on the resulting window statistics is expected to be minimal. The vegetative growing season was defined as the period from 1 March (approx. DOY 60) to 30 October (approx. DOY 303) each year based on long-term observation averages. However, to account for the potential influence of pre-emergence climatic conditions (e.g., dormancy release) on spring phenology, the meteorological analysis period for the sliding-window search was extended to cover DOY 15 to DOY 303. All DOY values used in this study are calculated relative to January 1st of the current year. Any window that would theoretically extend beyond the end of the defined analysis period (DOY 303) was automatically truncated or excluded from the search space.

### 2.3 Sliding window analysis of major climatic drivers of the Lanzhou lily phenology

To quantitatively assess the temporal sensitivity of Lanzhou lily phenophases to key climatic variables—including mean air temperature, sunshine duration, and precipitation—a sliding-window correlation analysis (Fu et al., 2019a; Zhang et al., 2025; Li et al., 2023) was employed to identify the optimal climatic response windows. This method systematically shifts a predefined time window along the growing season and computes Pearson correlation coefficients between the mean or accumulated values of each climatic variable within the window and the observed phenological dates. For each climatic variable, the analysis was conducted independently across a range of candidate start dates and window lengths, acknowledging that different environmental factors may influence phenological development during distinct preceding periods. For each variable, the window exhibiting the strongest statistically significant correlation was identified as its climatic sensitivity window. Because adjacent windows overlap substantially and are therefore not statistically independent, the procedure should be interpreted as a continuous sensitivity scan—designed to locate biologically plausible response periods—rather than a series of independent hypothesis tests.

The analysis involved four major steps: (1) Window parameterization: the starting date of each window was defined within DOY 15–270, and window lengths ranged from 15 to 100 days (15, 20, 25, 30, 40, 50, 60, 70, 80, 90, and 100) to comprehensively capture potential sensitivity periods for different phenophases. The early start date (DOY 15) was selected to ensure that climatic drivers of dormancy release and bulb activation prior to the formal growing season were captured. The window duration ranged from 15 to 100 days: the lower limit was set to 15 days to filter out short-term weather noise, while the upper limit of 100 days ensured that the climatic signals remained focused on physiologically relevant periods, avoiding the dilution of signals across multiple seasons. Crucially, for each phenophase, the search space was constrained such that the window end date could not exceed the actual observed date of that phenophase in any given year. This prevents the inclusion of post-event meteorological data in the sensitivity analysis of preceding stages. (2) Computation of climatic indicators: for each window, mean air temperature (°C) was calculated as the average of daily temperatures, sunshine duration (Ssd) was calculated as the mean of daily sunshine-duration values (unit: h d^-^¹), and precipitation (Pre) was calculated as the cumulative sum over the window (mm). Thus, Ssd_a–b represents the mean daily sunshine duration during DOY a–b. (3) Correlation analysis: the Pearson correlation coefficient (r) was used to quantify the linear relationship between each climatic factor and the timing of phenological stages across all moving windows. The statistical significance of r was evaluated using a two-tailed t-test (*p* < 0.05), and larger |r| values indicated stronger climatic control during the corresponding period. (4) Identification of optimal response window—the time interval exhibiting the maximum absolute correlation coefficient (|r|_max_) with statistical significance (*p* < 0.05) was defined as the optimal climatic response window, representing the period of greatest climatic sensitivity for each phenophase. To account for the potential inflation of nominal significance due to multiple testing, the ‘optimal window’ was identified not only by the maximum absolute correlation coefficient (|r|_max_) but also by its position within a stable significance cluster. An optimal window was considered robust only if it was part of a contiguous region of pixels with *p* < 0.05, thereby minimizing the risk of selecting chance extrema.

All analyses were conducted in Python 3.12, using the pandas (v2.3), NumPy (v1.26), and SciPy (v1.15) libraries for data processing and statistical analysis. Visualization was performed using Matplotlib (v3.9) and Seaborn (v0.13).

### 2.4 Multiple linear regression analysis

Multiple Linear Regression (MLR) (Li et al., 2015; Gu et al., 2022) was employed to quantify the combined effects of climatic variables on the phenological stages of the Lanzhou lily. This approach allows the relative contributions of temperature, sunshine duration, and precipitation to be evaluated simultaneously, while accounting for potential covariance among these climatic drivers. Single-variable regressions were not adopted as the main analytical framework because they cannot separate the shared effects of correlated predictors. Although phenological responses may exhibit nonlinear behavior, the dataset consists of 30 annual observations, and a linear model provides a parsimonious and interpretable framework for identifying the dominant climatic controls. The regression coefficients were estimated using the Ordinary Least Squares (OLS) method, which minimizes the sum of squared residuals between observed and predicted values. The analytical workflow consisted of five key stages: data preparation, variable selection, model fitting, diagnostic evaluation, and predictive validation. All analyses were performed in SPSS 26.0 (IBM Corp., Armonk, NY, USA) using the stepwise backward elimination procedure. This approach began with a full multiple linear regression model including all candidate climatic predictors (temperature, sunshine duration, and precipitation) identified from the sliding-window analysis. The full model can be written as:


$$P_{i}=\beta_{0}+\beta_{1}Tem_{i}+\beta_{2}Ssd_{i}+\beta_{3}\mathrm{Pr}e_{i}+\epsilon_{i}$$

where ${\text{P}}_{\text{i}}$ is the phenological date (DOY) in year *i*, ${\text{Tem}}_{\text{i}}$represents the mean temperature within the identified climatic window, ${\text{Ssd}}_{\text{i}}$ is the sunshine duration, ${\text{Pre}}_{\text{i}}$ denotes precipitation, ${\text{β}}_{0}$ is the intercept, ${\text{β}}_{1}$, ${\text{β}}_{2}$, and ${\text{β}}_{3}$ are regression coefficients, and ${\text{ϵ}}_{\text{i}}$ is the random error term.

Iterations continued until all remaining predictors were statistically significant (*p* < 0.05). For each model, diagnostic indicators—the coefficient of determination (R²), ANOVA, regression coefficients, and variance inflation factor (VIF)—were used to assess model fit and multicollinearity. Standard assumptions of residual normality, independence, and homoscedasticity were verified to ensure model validity. Particular care was taken to avoid overfitting and to ensure interpretability of the regression coefficients. The robustness of the multiple linear regression models was evaluated using the Durbin-Watson test for residual autocorrelation and the Variance Inflation Factor (VIF) for multicollinearity. Only models with no significant autocorrelation (D-W values near 2.0) and low collinearity (VIF< 5) were reported. Key statistical outputs, including Model Summary, ANOVA Table, Coefficient Table, Variable Removal Process, Residual Histogram, Residual P-P Plot, and Residual Scatter Plot, are presented in **Appendix Tables A3–A6**; **Appendix Figures A1–A3**.

1. ES: the regression model aimed to identify the major climatic factors controlling the onset of shoot emergence. Based on the results of the sliding-window analysis, two variables were selected as key predictors: Tem_54–69 (mean temperature during DOY 54–69) and Ssd_29–44 (Sunshine duration during DOY 29–44). These parameters represent the early pre-emergence period, during which temperature accumulation and solar radiation play critical roles in dormancy release and bud activation.
2. FBS: the regression analysis examined how pre-flowering climatic conditions influenced the timing of flowering. Two predictor variables were incorporated: Tem_98–108 (Mean temperature during DOY 98–108) and Ssd_162–177 (Sunshine duration during DOY 162–177) according to the results of the sliding-window analysis. These windows correspond to the period of floral induction and mid-season light availability, respectively.
3. SSS: the model focused on identifying late-season climatic controls on above ground organ senescence. Two key predictors were retained from the sliding-window correlation results: Tem_221–241 (Mean temperature during DOY 221–241) and Ssd_222–252 (Sunshine duration during DOY 222–252). These variables represent the terminal growth period, when heat accumulation and light exposure jointly regulate the transition from active photosynthesis to senescence.
4. GSL: MLR was used to assess the integrated climatic control over the entire phenological sequence. Three climatic variables were included as predictors: Tem_226–241 (Mean temperature during DOY 226–241), Ssd_222–252 (Sunshine duration during DOY 222–252) and Pre_245–260 (Precipitation during DOY 245–260). These indicators represent the late growing period and were selected to reflect the combined influences of temperature, radiation, and moisture availability on the duration of the active growth cycle.

## 3 Results

### 3.1 The trend of meteorological variables change in the Lanzhou region

Over the past three decades, the Lanzhou region has undergone a pronounced warming trend (**Table 1**). The mean growing-season temperature (T_m_) averaged 16.95 ± 0.13 °C, increasing significantly at a rate of 0.57 °C per decade (*p* < 0.001). The maximum temperature (T_max_) reached 35.587 ± 0.30 °C, exhibiting a notable warming rate of 0.71 °C per decade (*p* < 0.05). The mean sunshine duration (Ssd) was 1368.38 ± 15.33 h, declining markedly at a rate of 64.5 h per decade (*p* < 0.001). Meanwhile, the accumulated temperature above 0 °C (∑T_0_) averaged 3102.97 ± 23.00 °C, showing a significant upward trend of 103.74 °C per decade (*p* < 0.001). In contrast, the minimum temperature (T_min_), mean relative humidity (RH) and total precipitation (Pre) exhibited no significant long-term variation (*p* > 0.05).

**Table 1:** Trends in growing-season (March–October) meteorological variables in Lanzhou from 1995 to 2024.

Meteorological variables	Tem/°C (decade^-1^)	T_max_/°C (decade^-1^)	T_min_/°C (decade^-1^)	RH/% (decade^-1^)	Pre/mm (decade^-1^)	Ssd/h (decade^-1^)	∑ T_0_/°C (decade^-1^)
Mean values	16.95 ± 0.13	35.587 ± 0.30	-4.880 ± 0.40	57.45 ± 0.67	226.58 ± 10.61	1368.38 ± 15.33	3102.97 ± 23.00
Slope	0.57***	0.71*	0.17	-1.33	-9.94	-64.5***	103.74***
R²	0.53	0.15	0.01	0.10	0.02	0.46	0.53

Tem, T_max_, and T_min_ represent the mean, maximum, and minimum air temperatures, respectively; RH denotes relative humidity, Pre indicates precipitation, Ssd refers to sunshine duration, and ∑T_0_ represents the accumulated temperature above 0 °C. “Mean values” indicate the multi-year averages of each climatic variable, while “Slope” denotes the linear trend (per decade) estimated using ordinary least squares (OLS) regression. The coefficient of determination (R²) reflects the strength of the linear relationship between each variable and year. Asterisks indicate the significance of trend (*p < 0.05, ***p< 0.001), values without asterisk indicate non-significance.

### 3.2 Mean characteristics and temporal trends of key phenological parameters of the Lanzhou lily

**Figure 2:**
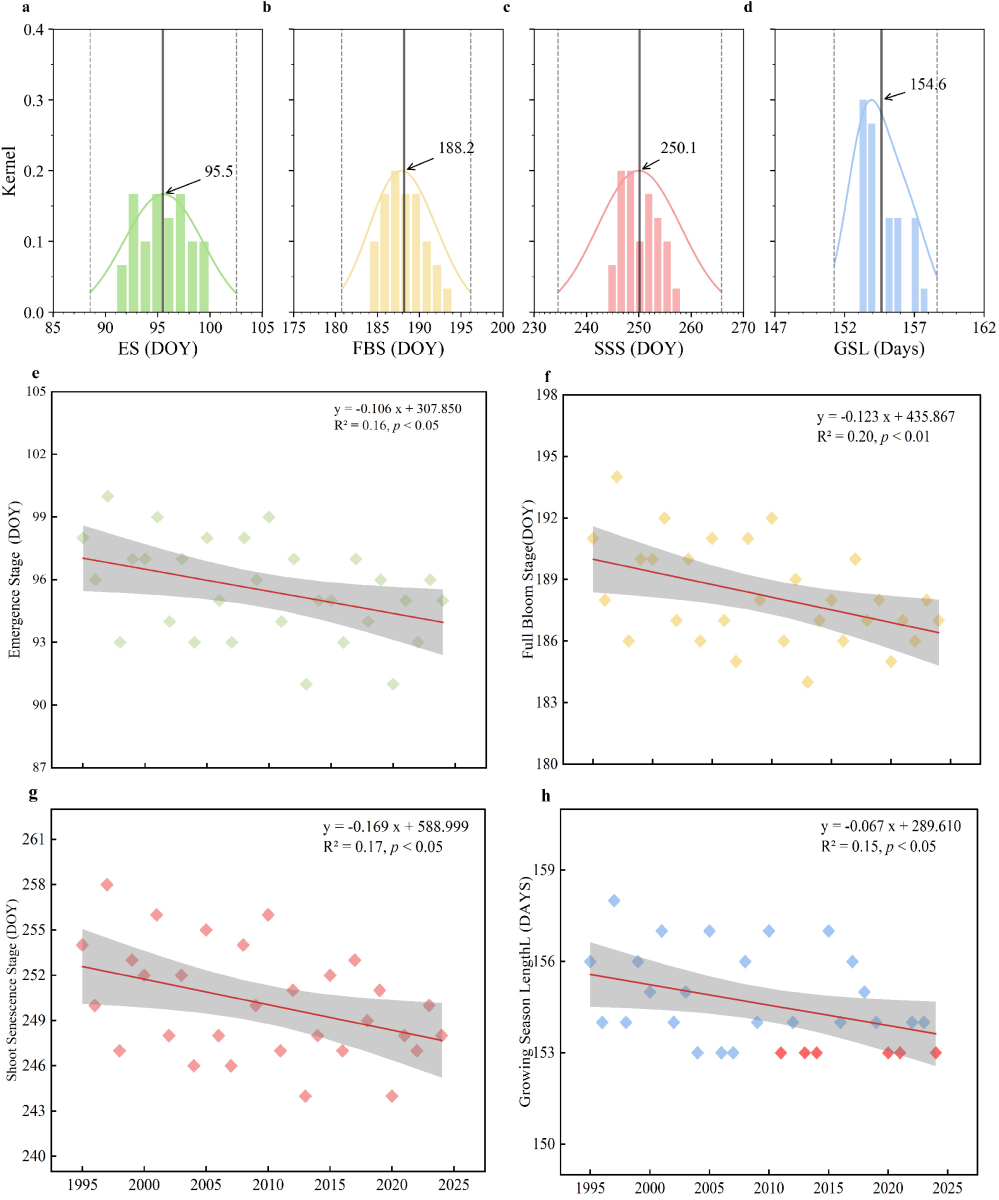
Kernel density estimates and temporal trends of phenological parameters of Lanzhou lily from 1995 to 2024. **(a–d)** show the kernel density distributions of the Emergence Stage (ES), Full Bloom Stage (FBS), Shoot Senescence Stage (SSS), and Growing Season Length (GSL). The x-axis represents phenological parameters, and the y-axis shows their relative frequency, reflecting the probability density across different years. The central black line and number denote the mean, black dashed lines indicate the 95% confidence interval, and the curve represents the kernel density estimate. **(e–h)** show the temporal trends of ES, FBS, SSS, and GSL, where the x-axis represents time and the y-axis the phenological parameters. The red line indicates the linear fit, gray shading shows the 95% confidence interval, and dots represent observed values. Red dots in **(h)** mark the minimum GSL value of 153 days.

The phenological stages of the Lanzhou lily, including the ES, FBS, SSS, and GSL, exhibited significant temporal shifts between 1995 and 2024 (**Figure 2**). On average, the ES occurred on DOY 95 (approximately 5 April) with a range of 9 days, showing a significant advancing trend at a rate of 1.06 days per decade (R² = 0.16, *p* < 0.05; **Figures 2a, e**). FBS occurred on DOY 188 (around 7 July), ranging 10 days and advancing at a rate of 1.23 days per decade (R² = 0.20, *p* < 0.01; **Figures 2b, f**). SSS occurred on DOY 250 (approximately 7 September), showing the largest interannual variability (range = 14 days) and a significant advancement of 1.69 days per decade (R² = 0.17, *p* < 0.05; **Figures 2c, g**). The GSL averaged 154.6 days, with a range of 5 days, and exhibited a shortening trend of 0.67 days per decade (R² = 0.15, *p* < 0.05; **Figures 2d, h**). Notably, the minimum GSL (153 days) occurred frequently after 2010, appearing six times within the study period (**Figures 2h**, red markers). These OLS trend estimates were broadly supported by modified Mann–Kendall tests and Sen’s slope, a non-parametric trend assessment, indicated consistent advancement for all phenophases, with significant trends for FBS, SSS and GSL (*p*_MK = 0.005, 0.026 and 0.031, respectively), whereas ES was marginal (*p*_MK = 0.056) (**Appendix Table A1**).

### 3.3 Regression analysis between phenological stages and meteorological factors

**Figure 3:**
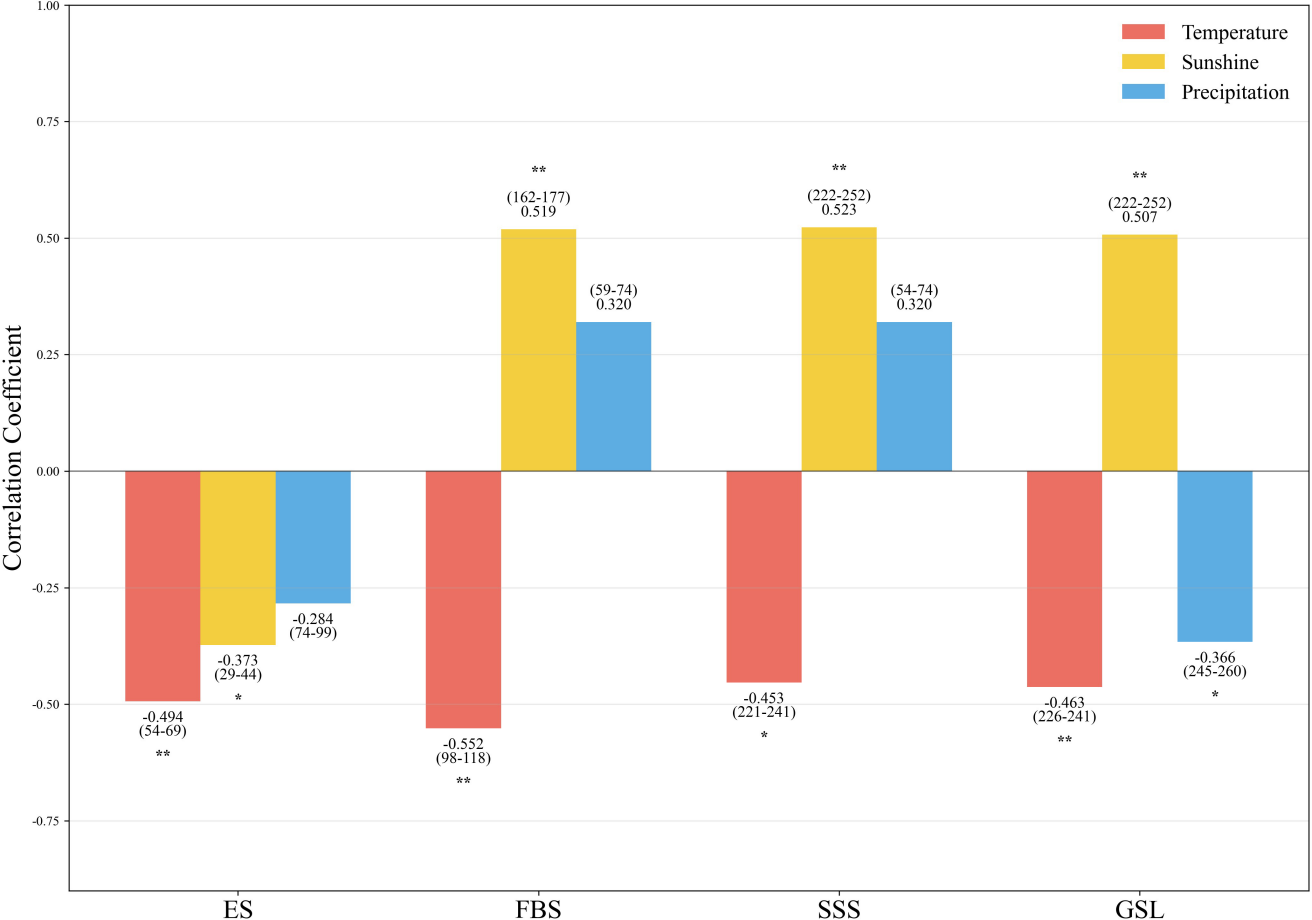
Correlation coefficient analysis between different phenological stages of the Lanzhou lily and climatic factors: The bar charts in the figure represent the correlation coefficient values, with the number ranges in parentheses indicating the corresponding DOY (Day of Year) periods. The significance levels are indicated by asterisks: **p < 0.01, *p < 0.05. A positive correlation coefficient indicates a positive correlation, while a negative value indicates a negative correlation.

The sliding-window analysis (**Figure 3**) revealed that mean temperature, sunshine duration, and precipitation exerted the most significant influences on the phenological development of the Lanzhou lily. Specifically, mean temperature during DOY 54–69 (Tem_54–69) exhibited a strong and significant effect on the ES. Both temperature during DOY 98–108 (Tem_98–108) and sunshine duration during DOY 162–177 (Ssd_162–177) significantly affected FBS. SSS was most strongly influenced by temperature during DOY 221–241 (Tem_221–241) and sunshine duration during DOY 222–252 (Ssd_222–252). Finally, GSL was jointly and significantly influenced by temperature during DOY 226–241 (Tem_226–241), sunshine duration during DOY 222–252 (Ssd_222–252), and precipitation during DOY 245–260 (Pre_245–260).

To ensure the reliability of the regression results, we conducted statistical diagnostics on the models. As shown in **Appendix Figures A1–A3**, the residuals of the four phenological models (ES, FBS, SSS, and GSL) satisfied the assumptions of normality (**Figures A1, A2**), as well as homoscedasticity and independence (**Figure A3**). These diagnostic results indicate that the multiple linear regression models employed in this study largely conform to classical statistical assumptions, yielding unbiased parameter estimates and valid statistical inferences.

**Figure 4:**
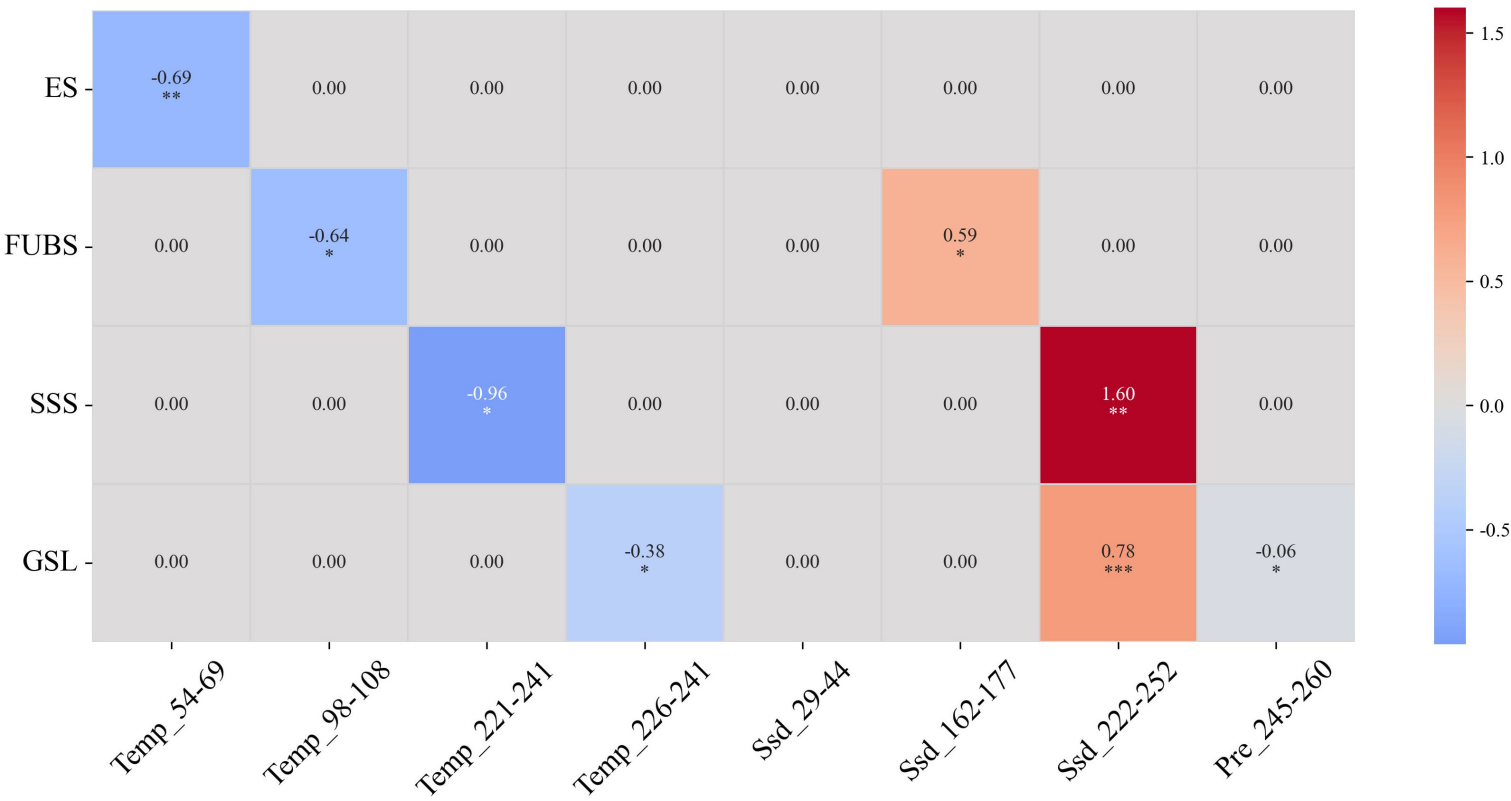
Correlation coefficient heatmap of the regression prediction models between phenological stages of Lanzhou lily and candidate climatic factors. The x-axis represents the climatic variables observed during specific time windows, for instance, Tem_54–69 refers to the mean temperature during DOY 54–69, Ssd_162–177 indicates the sunshine duration during DOY 162–177, and Pre_245–260 corresponds to the precipitation accumulated during DOY 245–260. The y-axis denotes the four key phenological indicators for ES, FBS, SSS, and GSL. And the significance levels of the correlation coefficients are marked as follows: *p < 0.05, **p < 0.01, ***p < 0.001. Cells without symbols indicate non-significant correlations.

The stepwise backward multiple linear regression analysis (**Figure 4**, **Appendix Table A2**) indicated that temperature was the dominant environmental driver of phenological advancement in the Lanzhou lily over the past three decades. Furthermore, the VIF for all predictors in the regression models were less than 5, indicating the absence of significant multicollinearity and ensuring the stability of the estimated coefficients. For ES, mean temperature was the primary factor (R² = 0.24, *p* < 0.01), with each 1°C increase advancing by 0.69 days. FBS was jointly regulated by temperature and sunshine duration (R² = 0.43, *p* < 0.01). A 1°C rise in mean temperature advanced the FBS by about 0.64 days, whereas each 1 h d^-^¹ increase in mean daily sunshine duration delayed it by approximately 0.59 days. Similarly, SSS was significantly influenced by both temperature and sunshine duration (R² = 0.39, *p* < 0.01). A 1°C rise in mean temperature advanced SSS by around 0.96 days, whereas each 1 h d^-^¹ increase in mean daily sunshine duration within the window delayed it by about 1.60 days. In contrast, GSL was jointly affected by temperature, sunshine duration, and precipitation (R² = 0.54, *p* < 0.01), suggesting an integrated climatic control on the overall phenological rhythm.

## 4 Discussion

Between 1995 and 2024, the ES, FBS, and SSS of the Lanzhou lily advanced significantly, while the GSL shortened by 0.67 days per decade. This overall “advancement–compression” pattern aligns with the global responses of temperate perennial plants to progressive warming (Piao et al., 2019; Zani et al., 2020; Li et al., 2025). The significant advancement of each phenophase can be primarily attributed to accelerated thermal accumulation and enhanced metabolic activity under increasing air temperature. In the Lanzhou region, the mean growing-season temperature rose by 0.57°C per decade and accumulated temperature above 0°C increased by 103.74°C per decade, both at highly significant levels (*p* < 0.001). Warmer spring conditions facilitate earlier dormancy release, bud differentiation, and shoot emergence from underground bulbs (Lee et al., 1996; Xu et al., 2021), thereby advancing the ES. Elevated temperatures also stimulate respiration and metabolic turnover, hastening senescence initiation (Chen et al., 2025; Yu et al., 2025) and compressing the overall growing season. As a cold-adapted bulbous species of the semi-arid Loess Plateau, the Lanzhou lily exhibits pronounced thermal sensitivity due to rapid physiological responses of its underground storage organs and its evolutionary adaptation to short-season heat utilization (Xu et al., 2021). And the spring emergence of Lanzhou lily may also be influenced by the winter dormancy release process. While the sensitivity windows identified in this study are primarily centered in early spring (March–April), the fulfillment of the chilling requirement is a physiological prerequisite for breaking endodormancy. Under the frigid winter conditions of Northwest China, the chilling requirement is currently fully met in most years, leaving spring forcing as the dominant driver of phenological advancement. However, temperature alone cannot fully explain the interannual variability in phenology, highlighting the importance of incorporating solar radiation and moisture factors for a more comprehensive understanding of phenological regulation.

Although both the FBS and SSS advanced over the past three decades, regression analyses revealed that increased sunshine duration delayed flowering and senescence, partially counteracting the thermal advancement effect. Each additional 1 h d^-^¹ in mean daily sunshine duration during the sensitivity windows (DOY 162–177 and DOY 222–252) corresponded to a delay of approximately 0.59 days for FBS and 1.60 days for SSS, respectively. This supports the “temperature-driven yet light-regulated” paradigm of plant development (Yanovsky and Kay, 2003; Searle and Coupland, 2004). Light acts not only as an energy source but also as a photoperiodic signal that modulates developmental transitions (Way and Montgomery, 2015). Adequate solar radiation enhances photosynthetic carbon accumulation and energy storage, prolonging vegetative activity and delaying senescence. However, excessive radiation may intensify transpiration stress (Wu et al., 2025), suggesting that the light–phenology relationship is context-dependent and seasonally constrained. For the Lanzhou lily, rising temperature accelerates phenological progress, Importantly, sunshine duration exhibits a significant long-term decline (-64.5 h decade^-^¹); therefore, at the trend scale, reduced sunshine would be expected to reinforce rather than offset warming-driven phenological advancement. The ‘countervailing’ role of sunshine identified here refers to interannual modulation: after accounting for temperature, years with anomalously higher sunshine during the sensitivity windows tend to delay flowering and senescence, partially buffering thermal advancement in some years. This dual mechanism demonstrates that the photothermal balance, rather than temperature alone, governs the seasonal rhythm of this bulbous perennial (Piao et al., 2019). Sunshine duration (Ssd) is not equivalent to photoperiod (astronomical daylength). Ssd primarily captures realized sunshine hours regulated by cloudiness and atmospheric transmissivity, and thus serves here as a proxy for regional-scale radiation background rather than a direct photoperiodic cue. The observed associations with phenology are therefore interpreted in terms of energy/carbon balance and potential evapotranspiration demands that may modulate development, while acknowledging that causal mechanisms require plot-level microclimate validation.

In the semi-arid climate of Lanzhou, water availability exerts an indirect but significant regulatory influence on phenology. The regression model for GSL (R² = 0.54, *p* < 0.01) identified precipitation during DOY 245–260 as an important late-season factor. Although precipitation did not significantly affect early stages (ES or FBS), its variability strongly influenced senescence timing. The local precipitation regime, concentrated between June and August, coincides with the flowering–senescence transition period, a phase when adequate soil moisture alleviates water stress and prolongs aboveground activity. Increased rainfall during this period enhances root absorption and photosynthetic maintenance, delaying senescence, whereas drought or poorly timed rainfall accelerates physiological decline and shortens the growing season. Such patterns have been widely reported across arid and semi-arid ecosystems (Fu et al., 2021; Zhang et al., 2023; Liu et al., 2024; Nie et al., 2025). Thus, while temperature remains the dominant driver, precipitation and radiation jointly buffer or amplify its phenological impacts, forming a dynamic triadic control of heat, light, and water that defines the phenological adaptability of the Lanzhou lily.

These results suggest that temperature and sunshine duration are strongly associated with variations in Lanzhou lily phenological timing within the analyzed dataset, several uncertainties and limitations should be acknowledged when interpreting the magnitude and generality of these responses. Because the sliding-window approach involves scanning multiple overlapping windows, the resulting sensitivity periods should be interpreted as exploratory indicators of climate–phenology coupling, and therefore serve as candidate climatic influence periods for subsequent regression-based attribution analysis. Despite statistically significant temporal trends, the moderate explanatory power of the regression models (R² ≈ 0.24–0.54) indicates that a substantial proportion of phenological variability remains unexplained, reflecting the inherently multi-factorial, lagged, and cumulative nature of phenological processes that cannot be fully captured by simple linear models. And both climatic drivers and phenological series exhibited significant long-term trends during the study period, the correlations identified might partially reflect shared temporal trends, future research should employ detrending techniques (e.g., first-order differencing) or control for ‘Year’ to further verify the stability of these climate-sensitivity windows. In addition to climatic forcing, non-climatic local factors—including groundwater availability, micro-topography, cultivation practices, and genetic background—may also play important roles but were not explicitly quantified in this study. In particular, Lanzhou lily is predominantly propagated through long-term vegetative reproduction, and if planting materials at the experimental site were not periodically renewed, genetic diversity may have gradually narrowed. Reduced genetic variation could enhance physiological sensitivity to temperature, potentially amplifying the observed climate-driven phenological responses relative to wild populations or farmer-maintained germplasm. Moreover, the assumption of linear relationships among climatic variables may oversimplify underlying ecological feedbacks, as threshold and saturation effects are frequently observed in phenological regulation (Pope et al., 2013; Meng et al., 2021), especially under extreme heat or excessive solar radiation. Finally, although the long-term record provides robust site-scale evidence, transferability of the identified climate-sensitive windows to broader regions should be treated as a testable hypothesis rather than a general rule. Validation across sites spanning gradients in elevation, slope aspect, soil texture, and management practices is needed to quantify how microclimate and cultivation context modulate the apparent sunshine-duration and temperature effects. A multi-site design combined with nearby station networks and plot-level microclimate measurements would allow disentangling regional background forcing and improve regional applicability.

Although direct yield data were not analyzed in this study, the observed phenological advancement carries significant implications for Lanzhou lily production. An earlier emergence extends the vegetative growth phase, which generally facilitates greater photosynthate accumulation and promotes bulb enlargement. Conversely, excessively early emergence may increase the risk of exposure to late spring frosts, potentially compromising bulb quality. Future research integrating long-term yield monitoring and biochemical profiling is required to quantify how shifts in climate-sensitivity windows dictate changes in biomass. The present analysis focused on a single medicinal bulbous species and did not situate Lanzhou lily within a broader comparative framework of temperate or high-altitude bulbous plants, such as *Lilium lancifolium* and related alpine lilies, or within the context of Northern Hemisphere high-altitude agricultural phenology. The absence of cross-species and cross-regional comparisons limits assessment of whether the pronounced temperature sensitivity observed here represents a species-specific adaptation or a more general ecological pattern. Future research should therefore expand spatial coverage, incorporate multiple germplasm sources and species, and employ more robust statistical frameworks—such as regularized regression (e.g., LASSO), cross-validation techniques, or machine-learning approaches—to mitigate selection bias and improve the mechanistic understanding and predictive capacity for the phenological responses of Lanzhou lily under continued climate warming.

## 5 Conclusions

Based on comprehensive analysis of phenological and climatic records for the Lanzhou lily (1995-2024), the following conclusions were drawn:

Over the past three decades, the mean growing-season temperature and the accumulated temperature above 0°C (∑T_0_) increased significantly. Correspondingly, the ES, FBS, and SSS advanced significantly at rates of 1.06, 1.23, and 1.69 days per decade, respectively, while the GSL shortened by 0.67 days per decade.

Temperature emerged as the dominant climatic driver, exerting significant negative effects on all four phenophases. The sensitive response windows extended from early March to late August, indicating a broad thermal control throughout the growing season. In contrast, sunshine duration exerted an opposite-signed interannual effect by delaying flowering and senescence in years with higher-than-average sunshine during the sensitivity windows, thereby modulating the temperature-driven advancement. Precipitation exerted a secondary but seasonally important influence, primarily modulating late-season senescence through moisture availability.

These findings highlight that interannual variation in Lanzhou lily phenology is jointly associated with temperature, sunshine duration, and precipitation, with temperature showing the strongest statistical influence across phenophases.

## Data Availability

The original contributions presented in the study are included in the article/**Supplementary Material**. Further inquiries can be directed to the corresponding authors.
