# Supplementary material for: Photothermal drivers and climate-sensitivity windows of Lanzhou lily (Lilium davidii var. unicolor) phenology across three decades of warming in Northwest China
Source: Front Plant Sci. 2026 Apr 17;17:1792696. doi: 10.3389/fpls.2026.1792696 (PMC13133685; doi:10.3389/fpls.2026.1792696)
Supplement: Supplementary file 1 [file DataSheet1.pdf]

Appendix Tables

Table A1. Trend robustness for the Phenology of the Lanzhou Lily (1995–2024) based on OLS and modified Mann–Kendall/Sen's slope

Phenology	OLS Slope	OLS p-value	OLS R^2	Modified MK p-value	Sen Slope
ES	-0.106	0.028	0.161	0.056	-0.111
FBS	-0.123	0.013	0.200	0.005	-0.120
SSS	-0.169	0.026	0.166	0.026	-0.167
GSL	-0.067	0.037	0.147	0.031	-0.053

Note: ES, emergence stage; FBS, full bloom stage; SSS, shoot senescence stage; GSL, growing season length. Slope units are days·year^{-1}. OLS Slope and OLS p-value are derived from ordinary least squares linear regression of each phenological metric against Year (1995–2024). OLS R^2 denotes the proportion of variance explained by the linear time trend. Modified Mann–Kendall (MK) p-values test the presence of a monotonic trend while accounting for serial autocorrelation; Sen's slope provides a robust non-parametric estimate of trend magnitude. Negative slopes indicate an advance (earlier timing) in day-of-year (DOY), and positive slopes indicate a delay.

Table A2. Regression Equation for the Phenology of the Lanzhou Lily

Phenology	Regression equation	R^2	*p*-value
ES	ES = 95.781 - 0.693 * Tem_54–69	0.24	<0.01
FBS	FBS = 190.345 - 0.641 *Tem_98–108 + 0.594 * Ssd_162–177	0.43	<0.01
SSS	SSS = 257.855 - 0.962* Tem_221–241 + 1.600 * Ssd_222–252	0.39	<0.01
GSL	GSL = 157.418 - 0.376 * Tem_226–241 + 0.783 * Ssd_222–252 - 0.060 * Pre_245–260	0.54	<0.01

Note: Regression equations for the four phenological stages of the Lanzhou lily in relation to key climatic factors. Tem, Ssd, and Pre represent mean temperature, sunshine duration, and precipitation, respectively. ES denotes the Emergence Stage, FBS denotes the Full Bloom Stage, SSS denotes the Shoot Senescence Stage, and GSL denotes the Growing Season Length. The numbers following each climatic factor indicate the optimal time windows (days of year, DOY) identified by the sliding-window analysis that showed the strongest correlation with each phenological parameter. For instance, Tem_54–69 refers to the mean temperature during DOY 54–69, Ssd_162–177 indicates the sunshine duration during DOY 162–177, and Pre_245–260 corresponds to the precipitation accumulated during DOY 245–260.

Table A3. Model summary of the stepwise multiple linear regression analysis of the four phenological stages of the Lanzhou lily

Model	R	R Square	Adjusted R Square	Std. Error of the Estimate	Change Statistics				
					R Square Change	F Change	df1	df2	Sig. F Change
ES_1	0.569	0.324	0.274	1.973	0.324	6.473	2	27	0.005
ES_2	0.494	0.244	0.217	2.049	-0.080	3.188	1	27	0.085
FBS_1	0.656	0.430	0.388	1.899	0.430	10.181	2	27	0.001
SSS_1	0.627	0.393	0.348	2.943	0.393	8.746	2	27	0.001
GSL_1	0.732	0.536	0.483	1.109	0.536	10.016	3	26	0.000

Note: Model summary of the stepwise multiple linear regression analysis for Emergence Stage (ES), Full Bloom Stage (FBS), Shoot Senescence Stage (SSS), and Growing Season Length (GSL) of the Lanzhou lily. R represents the correlation coefficient between the observed and predicted values. R Square and Adjusted R Square denote the proportion of variance in ES explained by the regression model before and after adjustment for the number of predictors, respectively. Std. Error of the Estimate indicates the standard deviation of residuals. R Square Change and F Change represent the incremental explanatory power and F-statistic resulting from the

inclusion of additional variables in the model, while df1 and df2 denote the degrees of freedom for the regression and residual, respectively. The significance of F Change (Sig. F Change) indicates whether the newly added variable significantly improves the model fit.

Table A4. Analysis of variance (ANOVA) summary for the stepwise multiple linear regression models of the four phenological stages of the Lanzhou lily

Model		Sum of Squares	df	Mean Square	F	Sig.
ES_1	Regression	50.397	2	25.198	6.473	0.005
	Residual	105.103	27	3.893		
	Total	155.500	29			
ES_2	Regression	37.986	1	37.986	9.051	0.006
	Residual	117.514	28	4.197		
	Total	155.500	29			
FBS_1	Regression	73.430	2	36.715	10.181	0.001
	Residual	97.370	27	3.606		
	Total	170.800	29			
SSS_1	Regression	151.549	2	75.774	8.746	0.001
	Residual	233.918	27	8.664		
	Total	385.467	29			
GSL_1	Regression	36.973	3	12.324	10.016	0.000
	Residual	31.993	26	1.231		
	Total	68.967	29			

Note: Analysis of variance (ANOVA) summary for the stepwise multiple linear regression models of Emergence Stage (ES), Full Bloom Stage (FBS), Shoot Senescence Stage (SSS), and Growing Season Length (GSL) of the Lanzhou lily. The table reports the partitioning of the total variance into regression and residual components for each model. Sum of Squares represents the explained and unexplained variability in ES; df denotes the degrees of freedom; Mean Square equals the Sum of Squares divided by df; F is the test statistic comparing the model variance to residual variance; and Sig. indicates the corresponding significance level (*p*-value).

Table A5. Regression coefficients for the multiple linear regression models of the four phenological stages of the Lanzhou lily

Model		Unstandardized Coefficients		Standardized Coefficients	t	Sig.	Collinearity Statistics	
		B	Std. Error	Beta			Tolerance	VIF
ES_1	(Constant)	101.471	3.208		31.628	0.000		
	Tem_54-69	-0.615	0.226	-0.438	-2.718	0.011	0.962	1.039
	Ssd_29-44	-0.845	0.473	-0.288	-1.786	0.085	0.962	1.039
ES_2	(Constant)	95.781	0.386		248.436	0.000		
	Temp_54-69	-0.693	0.230	-0.494	-3.008	0.006	1.000	1.000
FBS_1	(Constant)	190.345	3.661		51.994	0.000		
	Tem_98-108	-0.641	0.232	-0.426	-2.760	0.010	0.887	1.127
	Ssd_162-177	0.594	0.244	0.376	2.438	0.022	0.887	1.127
SSS_1	(Constant)	257.855	9.821		26.254	0.000		
	Tem_221-241	-0.962	0.417	-0.354	-2.305	0.029	0.951	1.052
	Ssd_222-252	1.600	0.554	0.444	2.890	0.008	0.951	1.052

GSL_1	(Constant)	157.418	3.717		42.354	0.000		
	Tem_226-241	-0.376	0.171	-0.308	-2.205	0.036	0.916	1.092
	Ssd_222-252	0.783	0.209	0.514	3.753	0.001	0.952	1.051
	Pre_245-260	-0.060	0.023	-0.363	-2.604	0.015	0.916	1.092

Note: Regression coefficients for the multiple linear regression models of the four phenological stages of the Lanzhou lily: Emergence Stage (ES), Full Bloom Stage (FBS), Shoot Senescence Stage (SSS), and Growing Season Length (GSL). B and Std. Error represent the unstandardized regression coefficients and their standard errors, respectively; Beta denotes the standardized coefficients reflecting the relative importance of predictors; t and Sig. correspond to the t-statistics and their significance levels (*p*-values). Tolerance and VIF (Variance Inflation Factor) are collinearity diagnostics, indicating the degree of multicollinearity among independent variables.

Table A6. Excluded variable from the stepwise multiple linear regression model for ES of the Lanzhou lily

Model		Beta In	t	Sig.	Partial Correlation	Collinearity Statistics		
						Tolerance	VIF	Minimum Tolerance
2	Ssd_29-44	-0.288	-1.786	0.085	-0.325	0.962	1.039	0.962

Note: Excluded variable from the stepwise multiple linear regression model for ES of the Lanzhou lily. Beta In represents the standardized regression coefficient of the variable at the point of exclusion, and t and Sig. correspond to the t-statistic and its significance level (*p*-value), respectively. Partial Correlation indicates the degree of association between the excluded variable and the dependent variable (ES) after controlling for the other predictors in the model. Tolerance and VIF (Variance Inflation Factor) are indicators of multicollinearity.

Appendix Figures

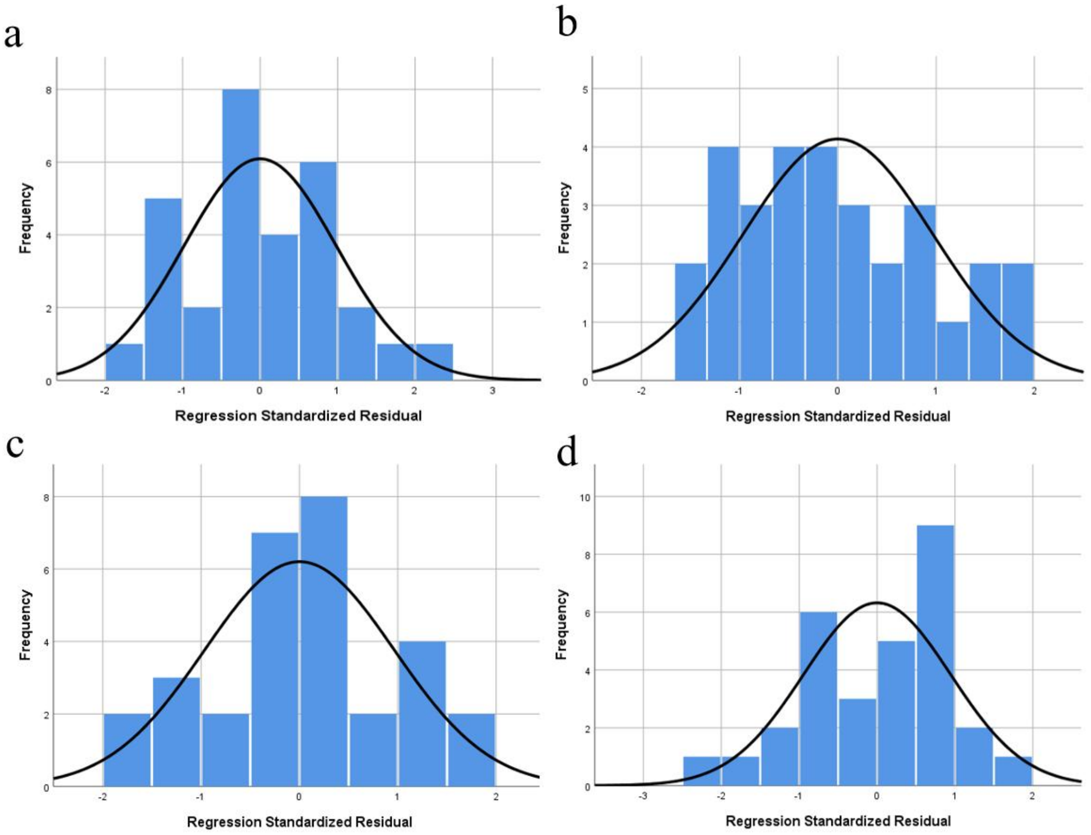

Fig. A.1. Histogram of regression standardized residual of ES(a), FBS(b), SSS(c), GSL(d). The x-axis represents the regression standardized residuals, and the y-axis indicates their frequency of occurrence. The blue bars represent the frequency distribution of standardized residuals, while the solid black curve indicates the fitted normal distribution. In all cases, the residuals exhibit an approximately symmetrical and bell-shaped pattern centered around zero, suggesting that the residuals follow an approximately normal distribution.

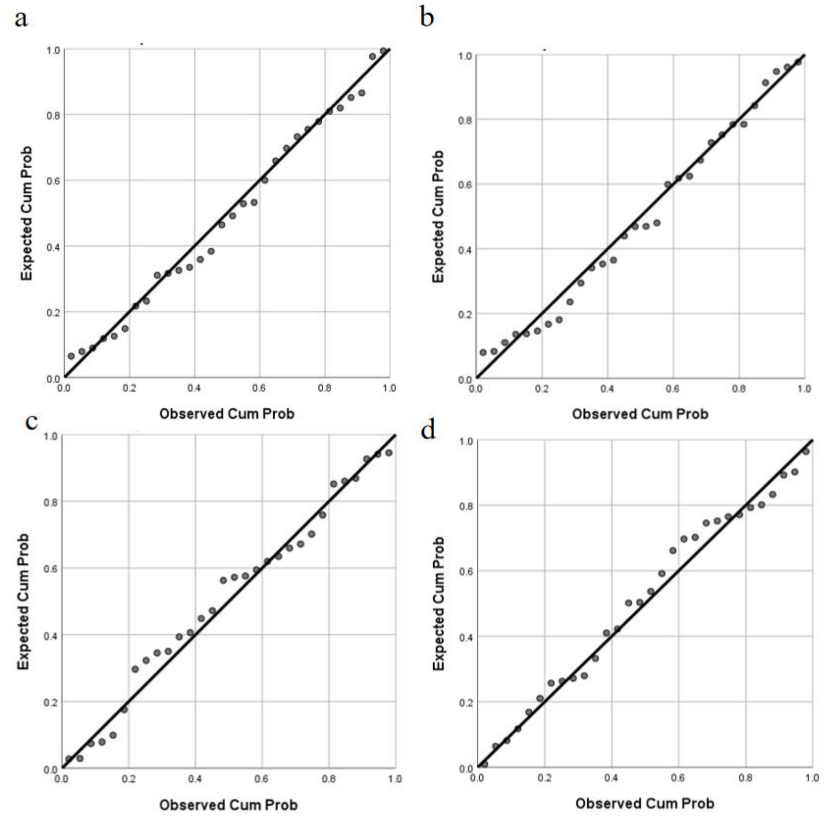

Fig. A.2. Normal P-P plot of regression standardized residuals for the model estimating the ES(a), FBS(b), SSS(c), GSL(d). The data points are closely aligned along the 45° reference line, indicating that the residuals follow an approximately normal distribution.

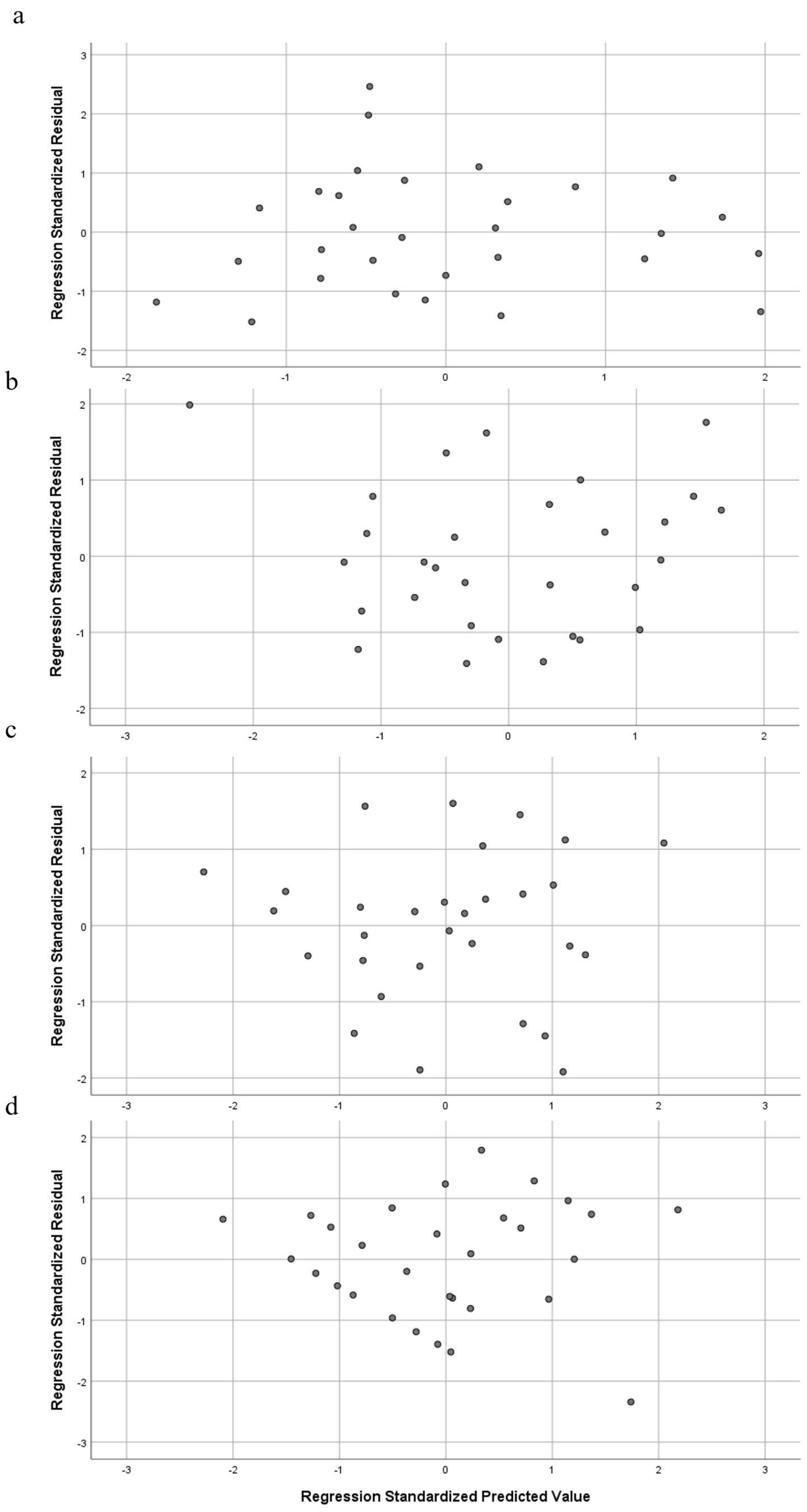

Fig. A.3. Scatter plots of regression standardized residuals versus standardized predicted values for the multiple linear regression models predicting the four phenological parameters: (a) ES, (b) FBS, (c) SSS, and (d) GSL. Each dot represents an individual observation. The residuals are randomly dispersed around zero without any discernible systematic pattern.
